# Remarkable Case of Spasm of the Glottis, with Unusual Symptoms*The caption is given by the Editor.

**Published:** 1845-05

**Authors:** Benjamin F. Eppes

**Affiliations:** Palestine, Sussex Co., Va.


*Remarkable Case of Spasm of the Glottis, with unusual Symptoms.** By Benjamin F. Eppes, of Palestine, Sussex Co., Va.

*Mr. Editor:*—I send you below a case which I consider uncommon, as you will see by comparing the symptoms with any known disease. I hope you will excuse me for omitting to give it any caption.

*History.* R. C. E., a child 15 months old, robust and healthy,

*The caption is given by the Editor.

was attacked at the age of six weeks (after suffering from its birth with catarrh,) with suspended respiration and lividity of countenance, which symptoms went off after some three or four hours of intense suffering. The child fattened and grew rapidly, became perfectly healthy, and deserved the appellation robust, as above remarked. On the evening of the 15th month he was attacked, without any premonitory symptoms; being as well, until the following symptoms were discovered, as he was ever known to be. He was put to bed at 9 o'clock, P. M.; my wife and self entered the room about 15 minutes past nine, and found him, as I thought, snoring and in a sound sleep. We thought no more of it until he was removed from the cradle to the bed; not then waking or ceasing the stertorous breathing, I was requested to examine him. On examination I found no symptoms of disease; the throat was not swollen externally, he had no fever, the skin natural, slight moisture on it. I recommended that the feet be tickled, thinking it would arouse him; but not so. I then had a pepper poultice applied to the throat, expecting it to arouse him. This failing, except for a very short time, (it cried a little and drank a mouthful or two of water, and relapsed again into its deep sleep.) There had nothing like spasm occurred up to this time, (about 11 o'clock.) There was no cough;—pressure around the throat, under the chin, and angle of the jaw, did not seem to produce pain; there was no lividity of countenance; the respiratory muscles seemed to be thrown into violent exertion to continue the function of respiration. The eyes were closed as in sleep, but a glutinous secretion seemed to come on which soon glued them together; the eye, when the ball was exposed, seemed to be natural; the pupil sensible to light.

I shall not attempt a diagnosis—as the only diseases which it may be mistaken for are acute laryngitis, spasmodic croup, œdema of the glottis, inflammation of the epiglottis, or spasm of the glottis—where all will find that none of these symptoms commence with coma.

*Treatment.*—I prescribed calomel, 4 grs.; ipecac. 4 grs. repeated every fifteen minutes until emesis was produced. The first dose caused a spasm of the glottis, the respiration not returning for a minute and a half, and was only produced by compressing the ribs and abdomen as in artificial respiration. The warm bath also was resorted to—lividity occurred, the respiration at last returned, and remained for about twenty minutes, when another spasm occurred, but not attended by so much lividity. Breathing restored by the same means. A flannel dipped in spirits of turpentine and applied to the throat produced redness, and yet no amelioration of symptoms. Dr. Field was now called in (a practitioner of great experience in diseases of children, having practised for twenty years extensively.) We bled the little patient from the arm to 3 or 4 ℥, but no abatement of the symptoms occurring, a large blister, covering the anterior of the thorax, and another on the back of the neck, were applied, both of which drew well, but the surface of each was pale; this was about 3 o'clock, the asphyxia occurring every twenty minutes from this time until about six o'clock, when the intermission was lengthened to half an hour, the longest time being two hours. I would here say, that moving the patient seemed to bring on the suspension of respiration. We attempted two or three times to give an emetic of antimonial wine, but it always produced suspended breathing. About 11 o'clock, A. M., there was spasm of the flexor muscles of the fore arms only; the eyes also rolled back and a distinct spasm occurred; five or six of these followed; the pulse was regular during the breathing but increasing in frequency; the cheeks commenced flushing after each suspension of respiration.

The child lived until twenty-five minutes past four. From about the first spasm (I think it was) a double flap occurred in the breathing. The last remedy used was the mustard bath, (excepting injections of tr. assafœtida, calomel, and castor oil.) The patient did not live five minutes after it was placed in the bath, which was not done until a spasm or asphyxia came on. To prevent any one from coming to the conclusion that these symptoms were produced by a foreign body in the larynx, I will state that the child ate his supper about seven o'clock, and sat up until nearly nine.

N. B.—I have sent this case to elicit information; there was no post mortem examination, but the right side to the median line became livid, while the other kept its natural colour; this was seen also in the blister.

*March* 15, 1845.

